# Non-targeted metabolomics aids in sex pheromone identification: a proof-of-concept study with the triangulate cobweb spider, *Steatoda triangulosa*

**DOI:** 10.1038/s41598-023-44948-0

**Published:** 2023-10-27

**Authors:** Andreas Fischer, Andrea C. Roman-Torres, Jane Vurdela, Yerin Lee, Nastaran Bahar, Regine Gries, Santosh Alamsetti, Hongwen Chen, Gerhard Gries

**Affiliations:** 1 Department of Biological Sciences, Simon Fraser University, Burnaby, BC V5A 1S6 Canada; 2 Department of General and Systematic Zoology, University of Greifswald, Greifswald, Germany; 3 Department of Chemistry, Simon Fraser University, Burnaby, BC V5A 1S6 Canada

**Keywords:** Chemical ecology, Chemical biology

## Abstract

Targeted metabolomics has been widely used in pheromone research but may miss pheromone components in study organisms that produce pheromones in trace amount and/or lack bio-detectors (e.g., antennae) to readily locate them in complex samples. Here, we used *non*-targeted metabolomics—together with high-performance liquid chromatography–mass spectrometry (HPLC–MS), gas chromatography-MS, and behavioral bioassays—to unravel the sex pheromone of the triangulate cobweb spider, *Steatoda triangulosa*. A ternary blend of three contact pheromone components [*N*-4-methylvaleroyl-*O*-isobutyroyl-l-serine (**5**), *N*-3-methylbutyryl-*O*-isobutyroyl-l-serine (**11**), and *N*-3-methylbutyryl-*O*-butyroyl-l-serine (**12**)] elicited courtship by *S. triangulosa* males as effectively as female web extract. Hydrolysis of **5**, **11** and **12** at the ester bond gave rise to two mate-attractant pheromone components [butyric acid (**7**) and isobutyric acid (**8**)] which attracted *S. triangulosa* males as effectively as female webs. Pheromone components **11** and **12** are reported in spiders for the first time, and were discovered only through the use of *non*-targeted metabolomics and GC–MS. All compounds resemble pheromone components previously identified in widow spiders. Our study provides impetus to apply *non*-targeted metabolomics for pheromone research in a wide range of animal taxa.

## Introduction

Sexually reproducing organisms commonly attract or locate mates through sexual communication signals. Signals may be uni-, bi- or poly-modal with visual, chemical, acoustic, vibratory, and tactile characteristics^1^. Pheromones are thought to be the oldest type of sexual communication signals^2^. They are chemicals, or blends of chemicals, released by a signaler that cause a response by conspecific signal recipients^3,4^. Pheromones are prevalent in many animal taxa^5^, including insects^6–8^, myriapods^9^, crustaceans^10^, fish^11^, and mammals^12^, and may be airborne or substrate-borne and be sensed by olfactory receptors^13^ or contact chemoreceptors^14^. Regardless of their physicochemical characteristics, pheromone components commonly occur in complex blends of analyte and are not easily located, isolated, and identified^15^.

Metabolomics entails the systematic identification and quantitation of metabolites, and their changes over time, in biological samples. Analytical techniques include, but are not limited to, gas chromatography—mass spectrometry (GC–MS), and high-performance liquid chromatography—mass spectrometry (HPLC–MS). The choice of analytical technique is based, in part, on chemical characteristics of target compounds and their abundance in samples. During GC–MS and HPLC–MS analyses, chemicals are separated and broken into mass fragments (ions), resulting in a mass spectrum for each sufficiently abundant compound and in a total ion chromatogram (TIC) that is created by summing up intensities of all mass spectral peaks belonging to the same scan^16^. Mass spectra then provide information about the molecular structure of compounds^16^.

**Figure 1:**
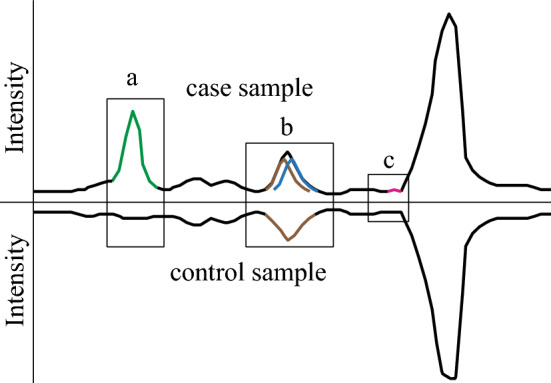
Graphical comparison of total ion chromatograms of a hypothetical case sample (upper trace) and a control sample (lower trace). (**a**) A unique compound (green) in the case sample is absent in the control sample. (**b**) A novel compound (blue) in the case sample is masked—and thus easily overlooked—by a compound (brown) present in both samples. (**c**) A unique trace compound (red) in the case sample might not be visually detected.

Comparative metabolomics in pheromone identification research compares analytes obtained from animals that were capable (e.g. sexually mature), or not (e.g. sexually immature), of producing pheromone^15^. Indeed, comparative metabolomics has been used to identify the first ever spider pheromone^17^. Traditional *targeted* metabolomics compares peaks between TICs and focuses on peaks for pheromone identification that are visually unique in one type of analyte (Fig. 1a)^18,19^. However, exclusive focus on visually unique peaks may miss pheromone components that co-elute with non-pheromonal compounds (Fig. 1b) or occur at trace quantities (Fig. 1c).

Pheromone research has focused on insects, with more than 3000 pheromones identified to date^6^. This remarkable progress is attributed to a landmark invention 53 years ago that combined gas chromatography with electrophysiology for insect pheromone analyses^20,21^. In these gas chromatographic-electroantennographic detection (GC-EAD) analyses, an insect’s antenna serves as a bio-detector to help locate candidate pheromone components in complex samples^22^. Moreover, in many insects, pheromone biosynthetic pathways and receptor sites are well understood^13,23^, and structural similarities of pheromones among congeners have expedited pheromone identifications^24^. In contrast, the chemical ecology of animals lacking antennae has hardly been studied, in part, because electrophysiological techniques were not applicable^6,15,25,26^.

Here, we applied *non*-targeted metabolomics (XCMS online)—together with GC–MS, HPLC–MS, and behavioral bioassays—to locate and identify both contact and mate-attractant pheromone components of a web building spider. XCMS online is a free and user-friendly metabolomics software (Scripps Research CA, USA)^27^ that enables analyses of data collected during mass spectrometric analyses such as GC–MS or HPLC–MS. Unlike *targeted* metabolomics, *non-targeted* metabolomics considers *all* detected ions and enables quantitative comparison of ions between samples. The software provides graphs and tables of ions as well as their relative abundance and retention times. This comprehensive approach reduces the probability of erroneously excluding peaks from analyses that are masked by other compounds (Fig. 1b) or occur at trace quantities (Fig. 1c).

Spiders have received little attention in chemical ecology research^6^. There are some 50,000 spider species but only 15 sex pheromones have been identified to date^6,15,25^, possibly because spiders lack antennae as pheromone bio-detectors (see above), and the search for pheromone receptors has met with limited success^26,28,29^. To assess how *non-untargeted* metabolomics can aid in spider pheromone research, we selected the triangulate cobweb spider, *Steatoda triangulosa*, a synanthropic, cosmopolitan spider inhabiting buildings^30,31^. We selected *S. triangulosa,* a tiny (3–6 mm) cob-web building spider^32^, because it belongs to a group of widow spiders (Latrodectinae) for which several pheromones have been identified^18,33–35^, anticipating that *non-targeted* metabolomics would help us find similar pheromone components in *S. triangulosa.*

**Figure 2:**
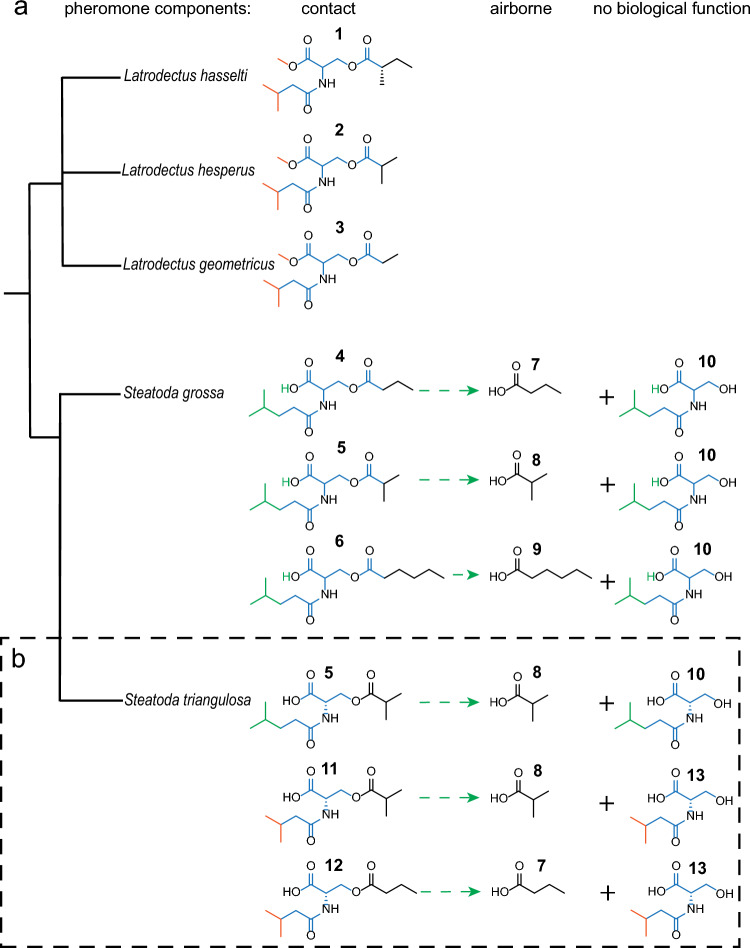
Phylogeny and comparison of pheromone components (contact & airborne) in widow spiders (Latrodectinae). (**a**) Previously known pheromone components of *Latrodectus hasselti*^18^, *L. hesperus*^33^, *L. geometricus*^35^, and *Steatoda grossa*^34^: *N*-3-methylbutyroyl-*O*-(*S*)-2-methylbutyroyl-l-serine methyl ester (**1**), *N*-3-methylbutanoyl-*O*-isobutyroyl-l-serine methyl ester (**2**), *N*-3-methylbutyroyl-*O*-isobutyroyl-l-serine-methyl ester (**3**), *N*-4-methylvaleroyl-*O*-butyroyl-l-serine (**4**), *N*-4-methylvaleroyl-*O*-isobutyroyl-l-serine (**5**), and *N*-4-methylvaleroyl-*O*-hexanoyl-l-serine (**6**). The contact pheromone components **4–6** of *S. grossa* hydrolyse at the ester bond and give to three airborne mate-attractant pheromone components [butyric acid (**7**), isobutyric acid (**8**), and hexanoic acid (**9**)], whereas the amide *N*-4-methylvaleroyl-l-serine (**10**), as another hydrolysis breakdown product, remains on webs and has no behavioural activity. (**b**) Pheromone components of *Steatoda* *triangulosa* identified in this study. The contact pheromone components *N*-4-methylvaleroyl-*O*-isobutyroyl-l-serine (**5**), *N*-3-methylbutyroyl-*O*-isobutyroyl-l-serine (**11**), and *N*-3-methylbutyroyl-*O*-butyroyl-l-serine (**12**) hydrolyse at the ester bond and give rise to two airborne mate-attractant pheromone components [butyric acid (**7**) and isobutyric acid (**8**)], whereas *N*-4-methylvaleroyl-l-serine (**10**) and *N*-3-methyl-butyroyl-l-serine (**13**) accumulate on webs. Blue-coloured parts of molecules are phylogenetically conserved, whereas green-coloured parts are unique to *Steatoda*. Orange parts are shared between *Latrodectus* spp. and *S. triangulosa*.

Within the Latrodectinae, females deposit contact pheromone components on their webs that elicit courtship by males upon contact. During courtship, males cut and bundle up sections of the female’s web, adding their own silk in the process^36^. Contact sex pheromone components have been identified for females of the redback spider, *Latrodectus hasselti* [*N*-3-methylbutyroyl-*O*-(*S*)-2-methylbutyroyl-l-serine methyl ester (**1**)]^18^, the western black widow spider, *L. hesperus* [*N*-3-methylbutanoyl-*O*-isobutyroyl-l-serine methyl ester (**2**)]^33^, the brown widow, *L. geometricus* [*N*-3-methylbutyroyl-*O*-propionyl-l-serine-methyl ester (**3**)]^35^, and the false black widow spider, *Steatoda grossa* [*N*-4-methylvaleroyl-*O*-butyroyl-l-serine (**4**), *N*-4-methylvaleroyl-*O*-isobutyroyl-l-serine (**5**) and *N*-4-methylvaleroyl-*O*-hexanoyl-l-serine (**6**)]^34^ (Fig. 2a). In *S. grossa,* contact pheromone components—web pH-dependently—hydrolyse at the ester bond and give rise to airborne mate-attractant pheromone components: butyric acid (**7**), isobutyric acid (**8**), and hexanoic acid (**9**) (Fig. 2a)^34^. *N*-4-Methylvaleroyl-l-serine (**10**), as another hydrolysis breakdown product, accumulates on webs and has no pheromonal activity^34^.

In this study, we applied four analytical tools—*non-targeted* metabolomics (XCMS online), HPLC–MS, GC–MS, and behavioural bioassays—to identify the contact and mate-attractant pheromone components of *S. triangulosa.* We demonstrate that these tools in combination, but not on their own, provided decisive analytical capability to unravel the complete pheromonal communication system of *S. triangulosa*.

## Methods

### Spider rearing and web collection

*Steatoda triangulosa* spiders used in experiments were the F1 and F3 offspring of females collected at the Black Widow Winery [Penticton, British Columbia (B.C.), Canada, 49.5467° N, 119.5698° W]. The spiders were reared in the insectary of the Burnaby campus of Simon Fraser University at 22 °C under a reversed light cycle (12:12 h). Spiderlings were kept individually in petri dishes (100 × 20 mm)^37^ fitted with moist cotton, and fed *Drosophila melanogaster* vinegar flies once per week. Virgin females and naïve adult males were randomly chosen for web-building and bioassays, respectively. As female spiders produce little silk per day, we extended the web-building period, allowing each female to build her web for seven days, instead of three days^34^, on a triangular prism (10 × 10 × 10 cm) of bamboo skewers (Goodcook, USA)^36^. Webs were collected using a methanol-cleaned glass rod and were extracted overnight in 25 µL/web of acetonitrile (ACN, 99%, Sigma-Aldrich, USA). All behavioral bioassays were run during the reversed scotophase (0900 to 1700).

### Analyses of web extracts by high-performance liquid chromatography—mass spectrometry (HPLC–MS)

Aliquots (2 µL) of web extracts of adult and subadult female *S. triangulosa* were analyzed, and compared, using coupled high-performance liquid chromatography—mass spectrometry (HPLC/MS). The Bruker maXis Impact Quadrupole Time-of-Flight LC/MS system consisted of an Agilent 1200 LC fitted with a Spursil C_18_ column (30 mm × 3.0 mm, 3 µm; Dikma Technologies, Foothill Ranch, CA, USA) and a Bruker maXis Impact Ultra-High Resolution tandem TOF (UHR-Qq-TOF) mass spectrometer. The LC/MS was operated with positive electrospray ionisation (+ ESI) at a gas temperature of 200 °C and a flow of 9 L/min. The nebuliser was set to 4 bar and the capillary voltage to 4200 V. The column was eluted with a 0.4 mL/min flow of a solvent gradient, starting with 80% water and 20% acetonitrile, and ending with 100% acetonitrile after 4 min. The solvent system contained 0.1% formic acid to improve the peak shape of compounds.

### Analyses of web extracts by gas chromatography—mass spectrometry (GC–MS)

Web extracts of adult virgin females were also analyzed by coupled gas chromatography—mass spectrometry (GC–MS), using an Agilent 7890B GC fitted with a DB-5 GC–MS column (30 m × 0.25 mm ID, film thickness 0.25 µm) and coupled to a 5977 A MSD. The injector port of the GC was set to 250 °C, the transfer line to 280 °C, the MS source to 230 °C, and the MS quadrupole to 150 °C. Helium was used as the carrier gas at a flow rate of 35 cm s^−1^. The following temperature program was used: 50 °C held for 5 min, a 10 °C/min increase to 280 °C (held for 10 min). Compounds were identified by comparing their mass spectra and retention indices with those of authentic standards that were synthesized in our laboratory. To improve chromatography of potential acids in web extracts, acids were transformed into trimethylsilyl-esters using BSTFA prior to analyses^38^.

### Analyses of web extracts by XCMS online

In search of further pheromone components, LC–MS analyses of web extracts of 10 adult females and 10 subadult female *S. triangulosa* were compared by XCMS online. The following parameters were used for the pairwise comparison: bw = 5, ppm = 10, peak width = c(2, 20), mzwidth = 0.01, and mzdiff = 0.01. Detected masses were sorted by “fold change” between the two groups and an arbitrary fold change of greater 35 × was selected as the threshold (see suppl. table).

### Behavioural testing of contact pheromone components—T-rod bioassays

#### General experimental design

The ability of web extract and of specific candidate contact pheromone components to induce courtship by male *S. triangulosa* was tested in T-rod bioassays, drawing on an established protocol^34,37,39^. The T-rod apparatus consisted of a horizontal beam (8 × 0.4 cm) and a vertical beam (8 × 0.4 cm) held together by labelling tape (3 × 1.9 cm, Fisher Scientific, Ottawa, ON, CA). A piece of filter paper (2 cm^2^) was attached to each distal end of the horizontal beam. The vertical beam of T-rods was inserted into plasticine (Craftsmart, Irving, TX, USA) placed in a tray (45 × 35 × 2.5 cm) partially filled with water to prevent spider males from escaping.

For each bioassay, three web equivalents in ACN or synthetic candidate pheromone components in ACN at three web-equivalents were applied to the randomly assigned treatment filter paper, whereas ACN was applied to the control filter paper. Using ACN as a corresponding control stimulus allowed us to account for potential solvent effects on male spider behaviour, and to demonstrate that male spiders do not court in response to ACN only. ACN was allowed to evaporate for 1 min before the onset of a 15-min bioassay. A randomly selected naïve male spider was then placed at the base of the vertical beam, and the time he spent courting on each filter paper was recorded. In response to the presence of female-produced or synthetic pheromone on a filter paper, the male engaged in courtship, pulling silk with his hindlegs from his spinnerets and adding it to the paper. Sensing contact pheromone, the male essentially behaves as if he were courting on the web of a female. Replicates of experiments as part of specific research objectives were run in parallel to eliminate day effects on responses of spiders. Treatment and control arms were alternated between replicates. T-rods and filter paper were discarded after use.

#### Specific experiments

The effect of web extract and of specific candidate contact pheromone components on courtship behaviour by male *S. triangulosa* was tested in three sets of T-rod bioassays. In set 1 (summer 2019), parallel experiments 1 and 2 (n = 20 each) tested web extract (three web equivalents in ACN) *versus* an ACN control (Exp. 1), and synthetic candidate contact pheromone component **5** *versus* an ACN control (Exp. 2). Synthetic **5** was tested at the same amount (387 ng) as present in three web extract equivalents. In set 2 (summer 2022), parallel experiments 3 and 4 (n = 20 each) tested web extract (three web equivalents in ACN) *versus* an ACN control (Exp. 3), and a blend of synthetic candidate contact pheromone components **5** (239 ng), **11** (8 ng) and **12** (11 ng) *versus* an ACN control. In set 3 (summer 2022), parallel experiments 5–8 tested **5**, **11** and **12** in ternary combination (Exp. 5; lure composition as in Exp. 4; total lure dose: 259 ng) and singly (Exps. 6–8), each at 259 ng.

### Chemical inferences and calculations of mate-attractant pheromone components

Drawing on previous findings that the contact pheromone components of *S. grossa* hydrolyse at the ester bond and give rise to acid mate-attractant pheromone components (Fig. 2b)^34^, we predicted that the contact pheromone components **5**, **11** and **12** of *S. triangulosa* would also hydrolyse and release butyric acid (**7**) and isobutyric acid (**8**) as mate-attractant pheromone components. However, we could not detect **7** and **8** in GC–MS analysis of silyl-ester derivatized web extract, and needed to estimate **7** and **8** based on amounts of the amide breakdown products **10** (*N*-4-methylvaleroyl-l-serine) and **13** (*N*-3-methylbutanoyl-l-serine) that originate from the hydrolysis and remain on webs (Fig. 2). With **10** quantified at 272 ng (1.34 mol) per web, and anticipating equal stoichiometric amounts of **8** and **10**, we decided to bioassay **8** at 124 ng per web. Moreover, with **13** not quantifiable in web extracts, we inferred the amount of **7** (5 ng) based on the 4% of **12** (which gives rise to **7**) in the 3-component contact pheromone blend.

### Sources of synthetic chemicals

Butyric acid (**7**) and isobutyric acid (**8**) (both 99% chemically pure) were purchased from Sigma-Aldrich (USA), *N*-4-methylvaleroyl-*O*-isobutyroyl-l-serine (**5**) was available from previous work in our laboratory^34^, and *N*-3-methylbutyroyl-*O*-isobutyroyl-l-serine (**11**), and *N*-3-methylbutyroyl-*O*-butyroyl-l-serine (**12**) were synthesized following the established synthesis of acylated serine derivates^18,33,34^. We report the ^1^H and ^13^C NMR spectra of **11** and **12,** as well as the mass spectra of esterified **11** and **12,** in the Supplementary Information.

### Behavioural testing of mate-attractant pheromone components—olfactometer bioassays

**Figure 3:**
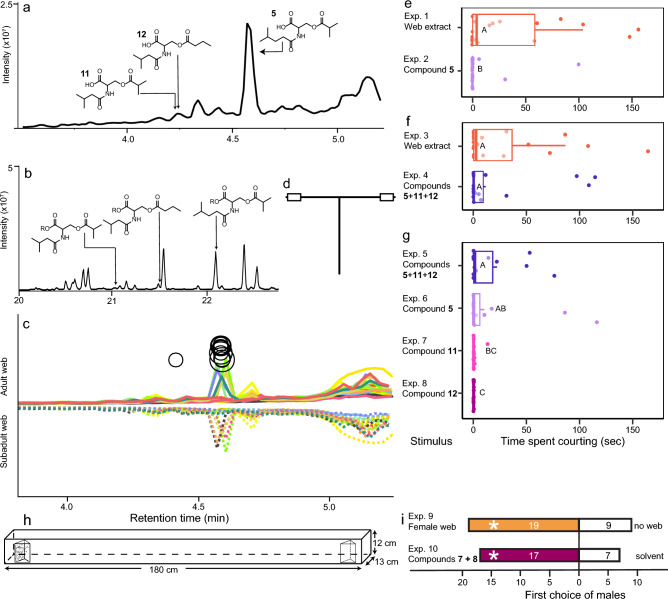
Chromatograms, experimental designs, and behavioural bioassay results. (**a**) Total ion chromatogram (TIC) of web extract of female *Steatoda triangulosa* analysed by high-performance liquid chromatography—mass spectrometry. (**b**) TIC of silyl ester-derivatized web extract of female *S. triangulosa* analysed by gas chromatography—mass spectrometry. (**c**) Comparative XCMS online Cloud Plots of web extracts of mature and immature female *S. triangulosa* (depicted by solid and dotted lines, respectively), with circles denoting a > 35-fold abundance increase of fragment ions in compounds; the larger the circle, the greater the fold-change of a particular ion. (**d**) T-rod bioassay apparatus. (**e**) Effects of female *S. triangulosa* web extract (Exp. 1) and contact pheromone component **5** (*N*-4-methylvaleroyl-*O*-isobutyroyl-l-serine) (Exp. 2) on courtship by *S. triangulosa* males. (**f**) Effects of female *S. triangulosa* web extract (Exp. 3), and a ternary blend of contact pheromone components **5**, **11** (*N*-3-methylbutyoyl-*O*-isobutyroyl-l-serine, and **12** (*N*-3-methylbutyroyl-*O*-butyroyl-l-serine) (Exp. 4), on courtship by *S. triangulosa* males. (**g**) Effects of contact pheromone components **5**, **11** and **12** presented in ternary combination (Exp. 5), and singly (Exps. 6–8), on courtship by *S. triangulosa* males. (**h**) Arena olfactometer with prisms carrying test stimuli. (**i**) Attraction of male *S. triangulosa* to webs of female *S. triangulosa* (Exp. 9), and to synthetic mate-attractant pheromone components **7** (butyric acid) and **8** (isobutyric acid) in arena olfactometers. In each of subpanels **e–g**, different letters indicate statistical differences between test stimuli across experiments (rank sum test; *p* < 0.05). In experiments 9 and 10 (subpanel **i**), the asterisk (*) indicates a significant preference for the test stimulus (binomial test; *p* < 0.05).

Attraction of males to webs of mature females spiders and to synthetic candidate mate-attractant pheromone components **7** (butyric acid) and **8** (isobutyric acid) was tested in still-air dual-choice olfactometers (winter 2022)^40^. Large Plexiglass arenas (180 cm × 12 cm × 13 cm, , Fig. 3h)^40^ lined with printer plot recorder paper (180 × 13 cm, Agilent, Santa-Clara, CA, USA) served as olfactometers. Two wooden prisms bearing (*i*) a female web or no web (Exp. 9, n = 30), or (*ii*) artificial (Halloween) web (45.05 ± 0.4 mg)^41^ treated with synthetic **7** (5 ng) and** 8** (124 ng) (Sigma-Aldrich) in ACN (75 µL), or an ACN control (75 µL) (Exp. 10, n = 30), were placed at opposite ends of the arena. For each bioassay, a single naïve male spider was placed into the centre of the arena, and allowed 30 min to approach and contact a prism, a behavioural response recorded as first choice. After each bioassay, the paper lining, webbing, and prisms were discarded. Treatment and control sides were alternated between replicates, and the same number of replicates was run for each of two experiments to eliminate potential day effects. Each male was tested only once.

### Statistical analysis

We analysed data using R (v. 4.3.1) and R-studio (v. 2303.06.0). A Wilcoxon Rank Sum test was used to compare the amount of time male *S. triangulosa* spent courting on filter paper treated with web extract (Exps. 1, 3) or a synthetic pheromone blend (Exps. 2, 4). A Kruskal–Wallis Rank Sum test with Benjamini–Hochberg correction was used to compare the amount of time male *S. triangulosa* spent courting on filter paper treated with a ternary pheromone blend or single components (Exps. 4–8). A one-sided binomial test^34^ with Benjamini–Hochberg correction for multiple testing was used to test effects of spider webs (Exp. 9), or a binary blend of synthetic mate-attractant pheromone components (Exp. 10), on attraction of male *S. triangulosa*.

## Results

### Analyses of web extracts by HPLC–MS, GC–MS and XCMS online

HPLC–MS analyses of web extract revealed a candidate contact pheromone component with fragment ions 296.1542 (M + Na), 274.1725 (M + 1) and 186.1179 (Fig. S1), matching the fragment ions and retention times of synthetic co-eluting **4** (*N*-4-methylvaleroyl-*O*-butyroyl-l-serine) and **5** (*N*-4-methylvaleroyl-*O*-isobutyroyl-l-serine)^37^. Coelution of **4** and **5** made it difficult to determine which compound was present but subsequent GC–MS analysis of esterified web extract determined that the compound was **5** (Fig. S3).

XCMS online confirmed the presence of **5** in web extracts (Fig. 3b). As female spiders progressed from subadults to adults, compound **5** ions 186.1179 and 274.1725 in web extracts increased 560-fold and 341-fold, respectively (Fig. 3b; Fig. S6). Another unknown compound (**X**), with retention time 4.42 min and fragment ion 260.1559, increased 37-fold (Fig. 3b).

GC–MS analysis of esterified extract in selected ion monitoring mode, searching for the 331.1815 ion of unknown **X**, revealed two isomers with retention time 21.05 min and 21.52 min. These isomers were identified as the trimethylsilyl-derivatives of *N*-3-methylbutyroyl-*O*-isobutyroyl-l-serine (**11**) (Fig. S4) and *N*-3-methylbutyroyl-*O*-butyroyl-l-serine (**12**) (Fig. S5).

### Behavioural testing of contact pheromone components—T-rod bioassays

Web extract of adult females elicited more sustained courtship behaviour than synthetic **5** as a single contact pheromone component (W = 272, p = 0.026; Fig. 3e, Exps. 1, 2), indicating the presence of additional contact pheromone components in web extracts. However, the ternary blend of contact pheromone components **5**, **10** and **11** was as effective as web extract in eliciting courtship behaviour by males (W = 215.5, p = 0.665; Fig. 3f, Exps. 3, 4), indicating that all essential components were present in the synthetic blend. The duration of male courtship on filter paper treated with **5**, **10** and **11** as a ternary blend, and singly, differed (χ^2^ = 14.52, df = 3, p < 0.001, Fig. 3g, Exps. 5–8). Statistically (but not numerically), **5** was as effective as the ternary blend, and more effective than **12** but not than **11**, in prompting and sustaining courtship by males (Fig. 3g).

### Behavioural testing of mate-attractant pheromone components—olfactometer bioassays

In arena olfactometers (Fig. 3h), prisms bearing webs of a female spider attracted more males than empty prisms (19 *vs* 9, N = 30, p = 0.043; Fig. 3i, Exp. 9), indicating the dissemination of airborne mate-attractant pheromone components from webs. Similarly, prisms bearing artificial (Halloween) web treated with synthetic mate-attractant pheromone components **7** and** 8** attracted more males than prisms bearing Halloween web treated with a corresponding solvent control (17 *vs* 7, N = 30, p = 0.043; Fig. 3i, Exp. 10), indicating that **7** and** 8** are the essential, and likely the only, mate-attractant pheromone components of *S. triangulosa*.

## Discussion

Our study provides proof of concept that a comprehensive analytical approach, entailing *non*-targeted metabolomics (XCMS online), HPLC–MS, GC–MS, and behavioural bioassays, was effective for unravelling the sex pheromone of the web-building spider *S. triangulosa*.

Whereas contact pheromone component **5** (*N*-4-methylvaleroyl-*O*-isobutyroyl-l-serine) would have been detected by conventional HPLC–MS, contact pheromone components **11** (*N*-3-methyl-butyryl-*O*-propionyl-l-serine) and **12** (*N*-3-methyl-butyryl-*O*-butyroyl-l-serine) were discovered only through the combined application of XCMS online, GC–MS, and behavioural bioassays. Through XCMS, we found that the fragment ion 260.1559 of an unknown compound (**X**) was 37-fold more abundant in web extracts of adult females than in web extracts of subadult females. GC–MS analyses of esterified extract, selectively scanning for the indicative ion of **X**, then revealed that **X** consisted of two isomers: **11** and **12**. In T-rod behavioural bioassays with male *S. triangulosa*, a ternary blend of synthetic **5**, **11**, and **12** was as effective as adult female web extract in eliciting courtship by males, indicating that all essential contact pheromone components were present in the synthetic blend.

Metabolomics has become a routine analytical tool to screen samples for the presence or relative abundance of compounds in ‘case’ samples relative to reference (control) samples^15,27^. *Non-*targeted metabolomics has been applied e.g. in studies of diet and health^43^, sport and exercise^44^, host-microbiota^45^, drug discovery^46^, plant metabololisms^47^, organismal responses to environmental toxicants^48^, and biomarker discovery in disease diagnosis^49^.

*Non*-targeted metabolomics as a pheromone research tool seems to have been used in only a recent single study^50^. Liu et al.^50^ hypothesized that the uropygial gland of ducks secretes chemicals that mediate sexual communication. Using LC–MS and principal component analyses, the authors found numerous metabolites in gland secretions, and noticed a gender-bias in metabolite secretions. Five compounds, in particular, were significantly more abundant in secretions of males than females: picolinic acid, 3-hydroxypicolinic acid, indolacetaldehyde, 3-hydroxymethyl-glutaric acid, and 3-methyl-2-oxovaleric acid. Although this male-biased abundance of compounds is helpful to select candidate sex pheromone components, their potential pheromonal signal function has still to be demonstrated in behavioural bioassays. In our *S. triangulosa* study, we applied *non*-targeted metabolomics, together with mass spectrometry and behavioural bioassays, to unravel new contact pheromone components and demonstrate their pheromonal signal function. To some extent, *non*-targeted metabolomics substituted for the analytical function of antennal bio-detectors that are lacking in spiders.

Prior pheromone chemistry knowledge of theridiid widow spiders (Fig. 2a)^18,33–35^ aided the identification of the *S. triangulosa* sex pheromone. *Steatoda triangulosa* and *S. grossa* share *N*-4-methylvaleroyl-*O*-isobutroyl-l-serine (**5**) as a contact pheromone component, but **5** is a minor component in *S. grossa* and the major component in *S. triangulosa* (Fig. 3). The two minor components of *S. triangulosa*—*N*-3-methylbutyroyl-*O*-propionyl-l-serine (**11**) and *N*-3-methylbutyroyl-*O*-butyroyl-l-serine (**12**)—are reported here for the first time as spider pheromone components. Notably, all currently known contact pheromone components of *Latrodectus* and *Steatoda* are acylated serine derivates with a conserved *N*-amide-*O*-ester core. Whereas pheromone components of female *Latrodectus* spp. have a methyl ester functionality and an *N*-4-methylvaleroyl-l-serine amide (**10**) rest, pheromone components of *S. grossa* and *S. triangulosa* have a free carboxylic acid—instead of a methyl ester—and either a *N*-4-methylvaleroyl-l-serine amide rest or a *N*-3-methyl-butyroyl-l-serine amide (**13**) rest (Fig. 2). All data combined reveal astounding structural similarity between theridiid pheromones, and imply a shared biosynthetic pathway. However, despite their common *N*-amide-*O*-ester serine motif, *Latrodectus* and *Steatoda* pheromones have unique characteristics that support the taxonomic assignment of these spiders to different genera^51^. While it has long been known that insect congeners produce structurally related pheromones, as shown in *Lymantria* moths^52–54^ and *Dendroctonus* bark beetle^55^, our study reveals an analogous phenomenon in two genera of web-building widow spiders.

In *S. grossa*, the contact pheromone components **4**, **5** and **6**—web pH-dependently—hydrolyze at the ester bond, giving rise to the airborne mate-attractant pheromone components butyric acid (**7**), isobutyric acid (**8**) and hexanoic acid (**9**) (Fig. 2)^34^. We have shown that this hydrolysis is likely catalyzed by a web-borne carboxyl ester hydrolase^34^, but did not search for this type of enzyme on webs of *S. triangulosa*, anticipating a similar pheromone breakdown mechanism. We inferred that the hydrolysis of both **5** (*N*-4-methylvaleroyl-*O*-isobutyroyl-l-serine) and **11** (*N*-3-methylbutanoyl-*O*-isobutyroyl-l-serine) would release isobutyric acid and that the hydrolysis of **12** (*N*-3-methylbutanoyl-*O*-butyroyl-l-serine) would release butyric acid as mate-attractant pheromone components (Fig. 2b). This inference proved correct because a binary blend of synthetic **7** and **8** attracted *S. triangulosa* males in arena olfactometers as effectively as female webs (Fig. 3i). The release of **7** and **8** as mate-attractant pheromone components is reminiscent of pheromonal communication in the spider *Linyphia triangularis*^17^, where both the dimer (*R*)-3-[(*R*)-3-hydroxybutyryloxy]-butyric acid and its breakdown monomer (*R*)-3-hydroxybutyric acid induce courtship by males, and where the monomer likely also attracts males^56^.

*Non*-targeted metabolomics was the key analytical tool to help us locate trace candidate pheromone components in complex *S. triangulosa* web extracts. However, many other analytical tools were still required for pheromone identification, with each tool contributing to the identification process. Moreover, various analytical steps of the identification process were carefully informed through behavioural bioassays with spiders. A similar analytical approach—albeit not yet including *non*-targeted metabolomics—was recently taken for the identification of the *S. grossa* sex pheromone^34^. Historically, however, bioassay-guided pheromone identifications, with the application of multiple sophisticated analytical tools, were pioneered in insects. The study unveiling the oviposition-deterring pheromone of the coccinellid beetle *Cheilosomones sexmaculata* exemplifies this type of multi-tool analytical approach^57^.

The integration of insect antennae as pheromone bio-detectors in GC-EAD analyses of complex odor samples^58^ has expedited insect pheromone identifications and enabled the discovery of trace pheromone components^59^. In contrast, pheromone research in animal taxa that lack antennae, such as spiders, or that rely on olfactory epithelia in nasal cavities and/or on vomeronasal organs for odor reception, such as mammals, reptiles and amphibians^5^, has progressed at a much slower pace^6^. Pheromones are known for only 15 spiders^15,34^, and for relatively few mammals^60^, reptiles^61^, and amphibians^62^. This paucity of progress may be attributed to the fact that olfactory receptors are not known for spiders^29,63,64^, or are deemed fully functional for vertebrates only in-vitro, which would require challenging preparations for pheromone research. However, there is now emerging evidence that metabolomics may be able—to some extent—to assume the analytical role of antennal bio-detectors in pheromone research. *Non*-targeted metabolomics was effectively applied to help select candidate pheromone components of ducks^50^ and spiders^this study^, both groups lacking antennae or equivalently effective bio-detectors for pheromone tracing. As a result, there is incentive now to apply *non*-targeted metabolomics for pheromone research in marine and terrestrial mammals, fish, birds, reptiles and amphibians, or even in signaling studies within and among plants.

In conclusion, we have applied *non-*targeted metabolomics, in combination with HPLC–MS, GC–MS, and behavioural bioassays, to unravel the sex pheromone of *S. triangulosa*. In this proof-of-concept study, we demonstrate that these four tools in combination, but not on their own, provide the analytical resolution to unravel the complete pheromonal communication system of a web-building spider. Our study provides impetus to take a similar analytical approach for pheromone research in other taxa that lack antennae or have odor receptors deemed fully functional only in-vivo.

### Supplementary Information

Supplementary Information 1.

Supplementary Information 2.

Supplementary Information 3.

## Data Availability

All data and code used in this manuscript have been added to the suppl. materials.
