# Supplementary material for: Non-targeted metabolomics aids in sex pheromone identification: a proof-of-concept study with the triangulate cobweb spider, Steatoda triangulosa
Source: Sci Rep. 2023 Oct 27;13:18426. doi: 10.1038/s41598-023-44948-0 (PMC10611747; doi:10.1038/s41598-023-44948-0)
Supplement: Supplementary file 3 — Supplementary Information 3. [file 41598_2023_44948_MOESM3_ESM.docx]

Non-targeted metabolomics aids in sex pheromone identification – a proof-of-concept study with the triangulate cobweb spider, *Steatoda triangulosa*

Andreas Fischer^1*^, Andrea C. Roman-Torres^1^, Jane Vurdela^1^, Yerin Lee^1^, Nastaran Bahar^1^, Regine Gries^1^, Santosh Alamsetti^1^, Hongwen Chen^2^, Gerhard Gries^1^

^1^Department of Biological Sciences, Simon Fraser University, Burnaby, British Columbia, V5A 1S6, Canada

^2^Department of Chemistry, Simon Fraser University, Burnaby, British Columbia, V5A 1S6, Canada

* Corresponding Author

Supplementary figures:


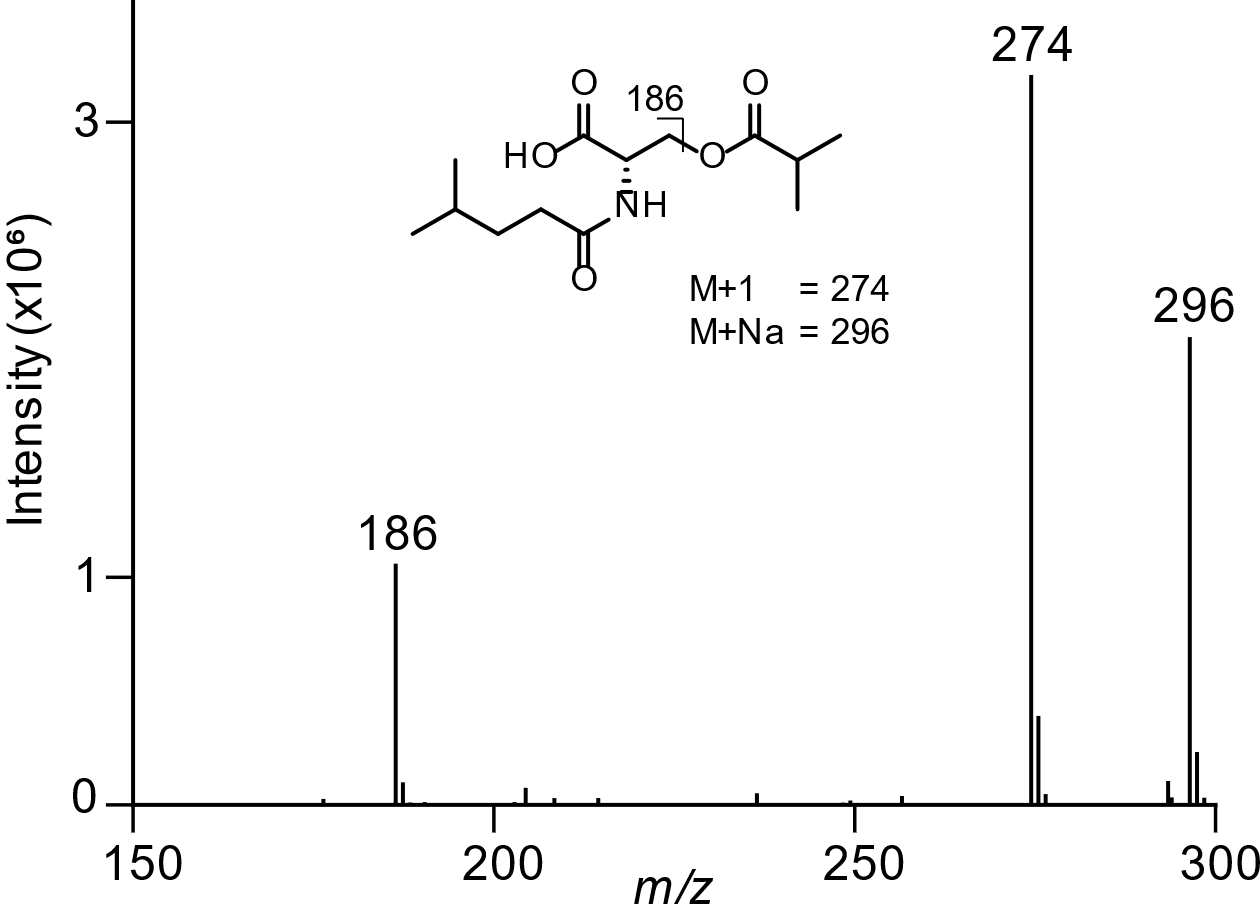


Supplementary Figure 1. Mass spectrum (positive electrospray ionization) of *N*-4-methylvaleroyl-*O*-isobutyroyl-L-serine extracted from webs of female *Steadoda triangulosa*.


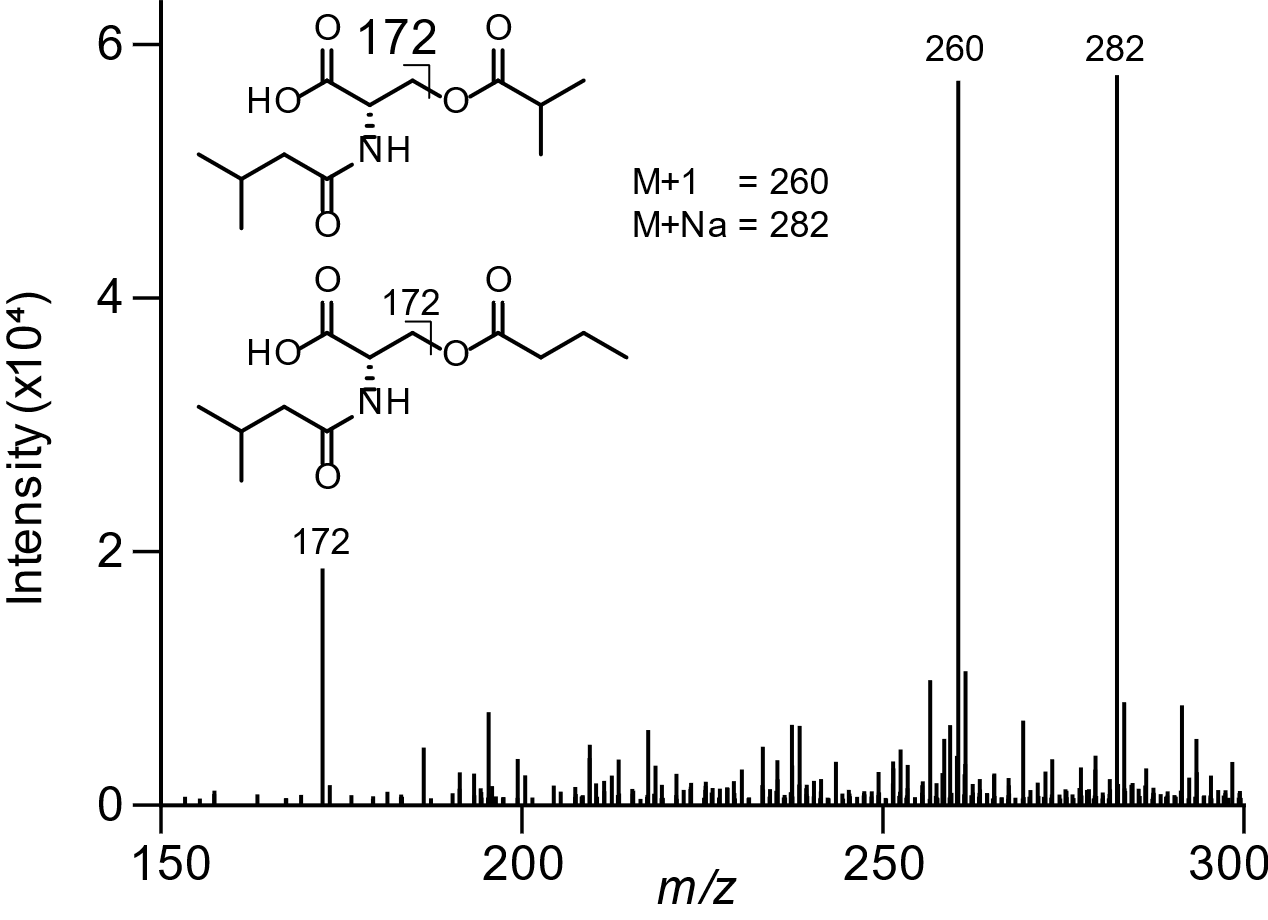


Supplementary Figure 2. Mass spectrum (positive electrospray ionization) of *N*-3-methylbutyroyl-*O*-isobutyroyl-L-serine and *N*-3-methylbutyorpyl-*O*-butyroyl-L-serine extracted from webs of female *Steadoda triangulosa*.


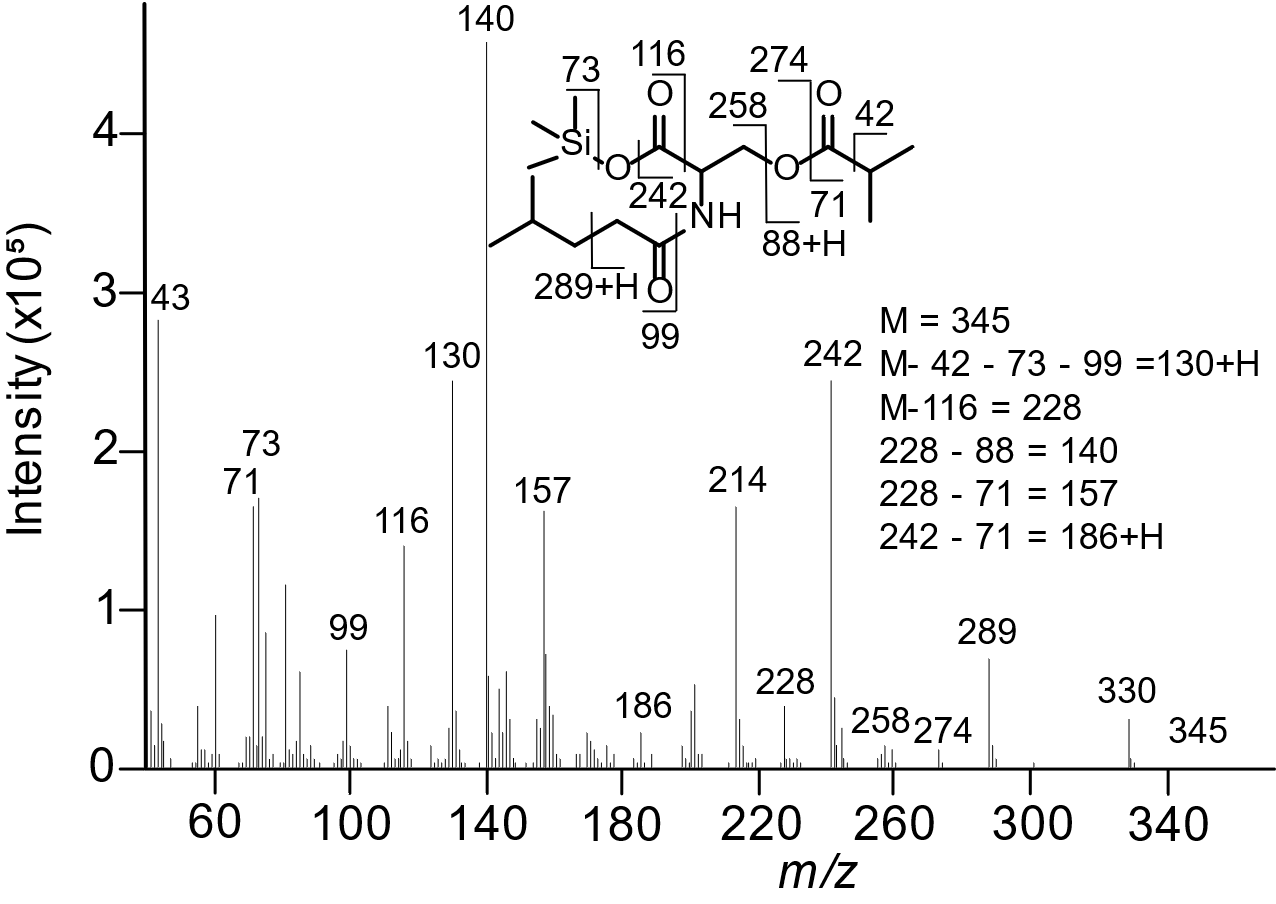


Supplementary Figure 3. Mass spectrum (electron ionization) of esterified *N*-4-methylvaleroyl-*O*-isobutyroyl-L-serine. The compound was extracted from webs of female *Steadoda triangulosa* and transformed into a trimethylsilyl-ester using BSTFA (*N*,*O*-bis(trimethylsilyl)trifluoroacetamide).


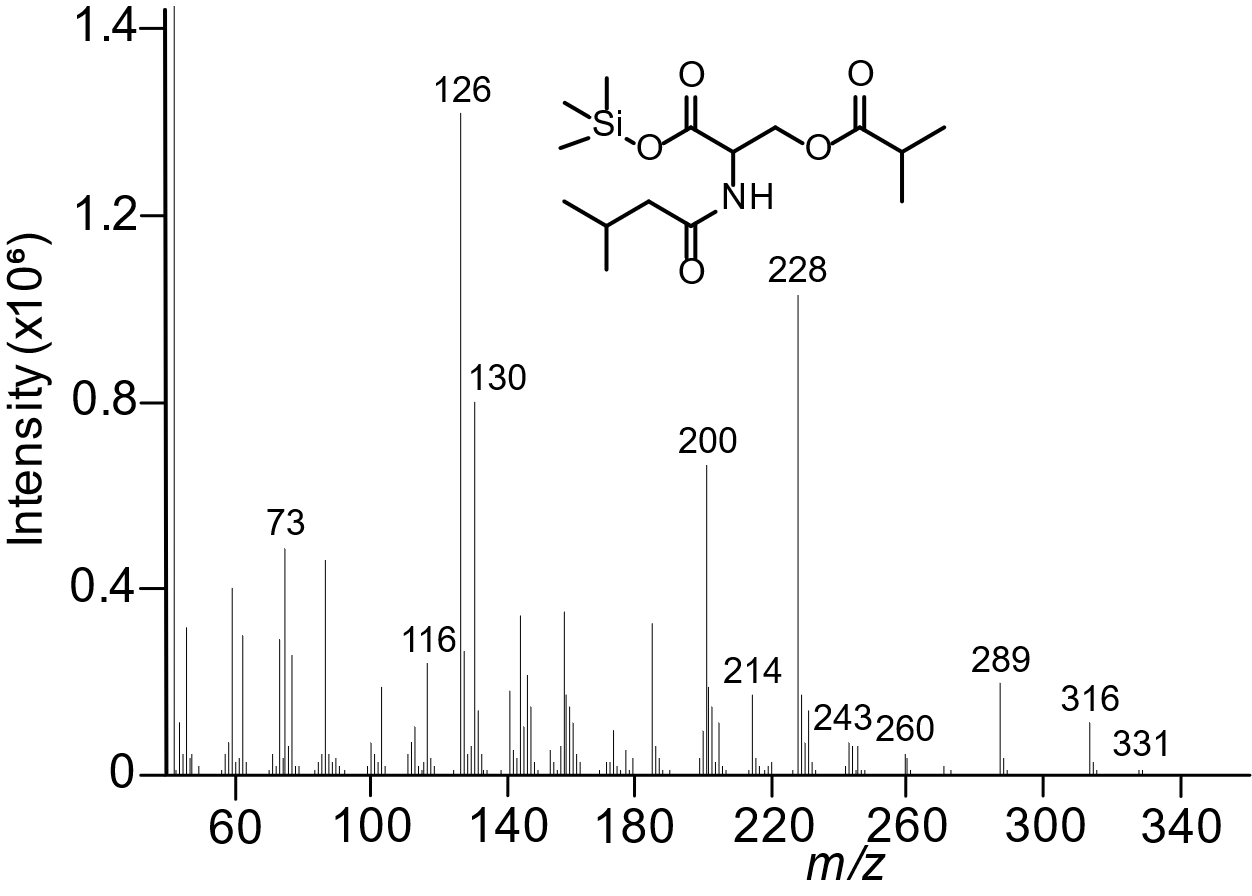


Supplementary Figure 4. Mass spectrum (electron ionization) of esterified *N*-3-methylbutyroyl-*O*-isobutyroyl-L-serine. The compound was extracted from webs of female Steadoda triangulosa and transformed into a trimethylsilyl-ester using BSTFA (N,O-bis(trimethylsilyl)trifluoroacetamide).


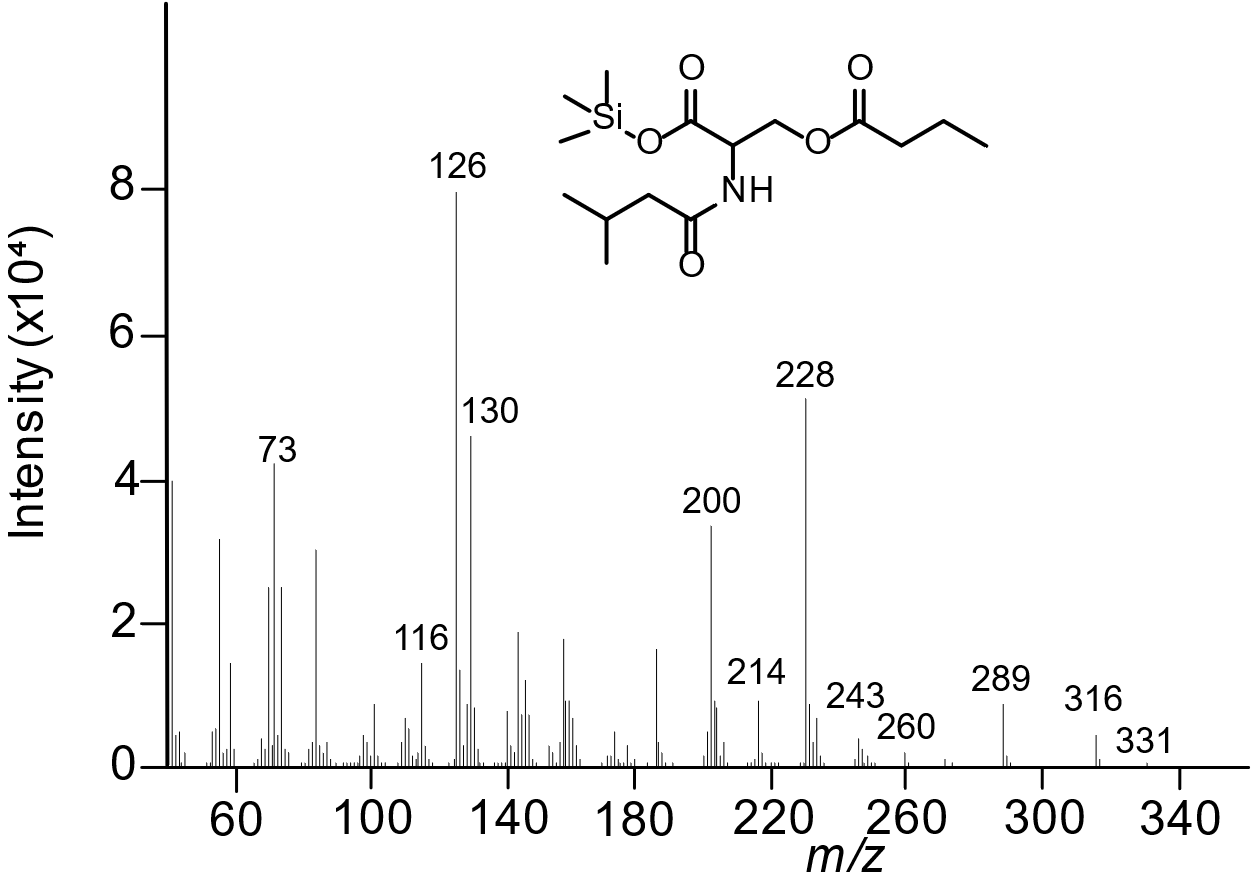


Supplementary Figure 5. Mass spectrum (electron ionization) of esterified *N*-3-methylbutyroyl-*O*-butyroyl-L-serine. The compound was extracted from webs of female *Steadoda triangulosa* and transformed into a trimethylsilyl-ester using BSTFA (*N*,*O*-bis(trimethylsilyl)trifluoroacetamide).


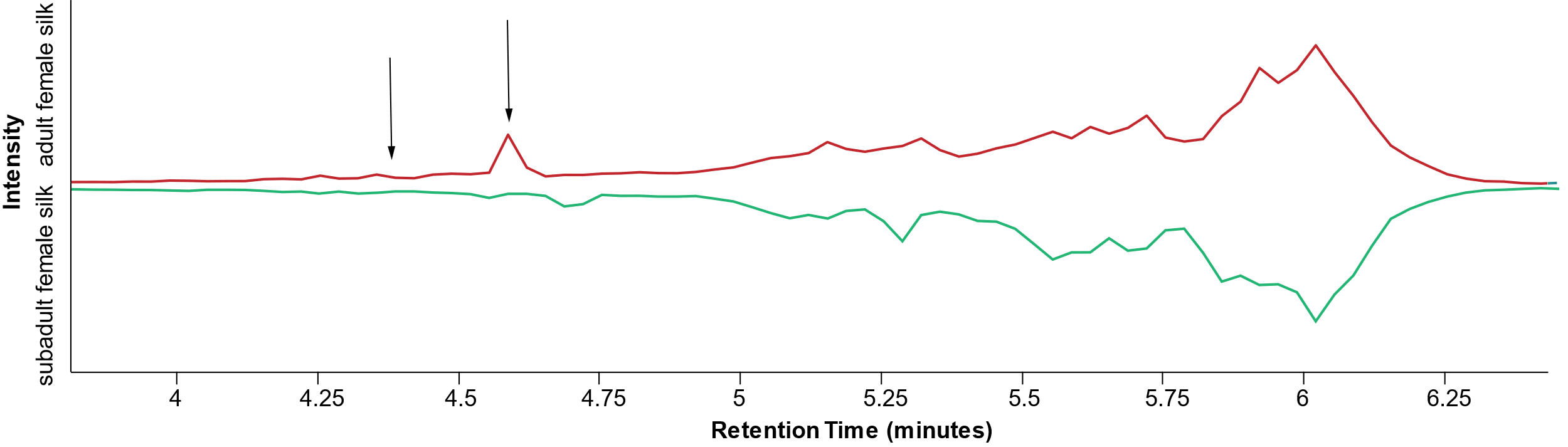


Supplementary Figure 6. Total ion chromatograms of web extract of adult (top) and subadult (bottom) female *Steatoda triangulosa* obtained by high-performance liquid chromatography - mass spectrometry. Arrows indicate the location of pheromone components.


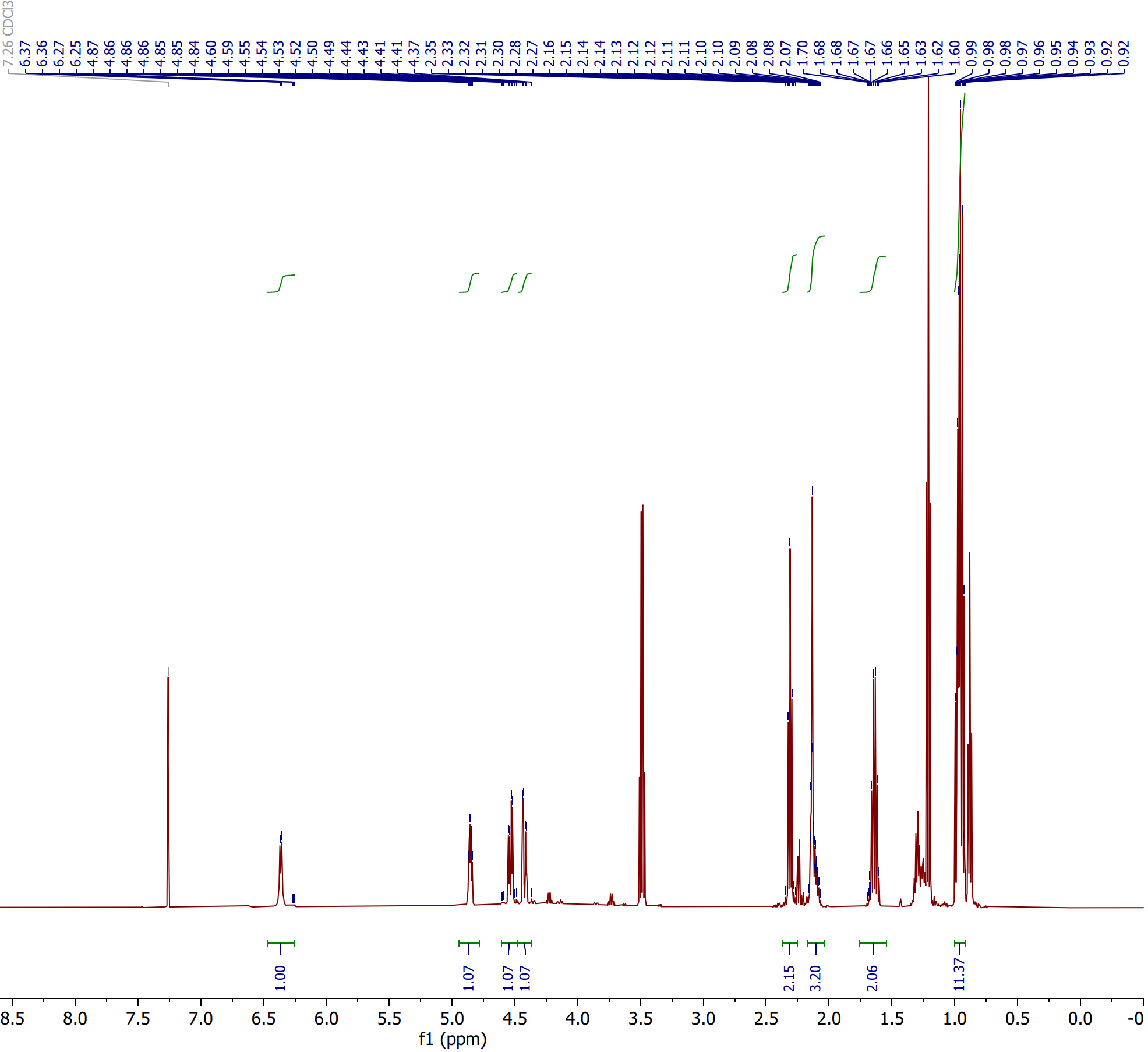


Supplementary Figure 7. ^1^H NMR spectrum of *N*-3-methylbutyryl-*O*-butyryl-L-serine produced by female *Steadoda triangulosa.* The ^1^H NMR spectrum was recorded on a Bruker Avance 600 equipped with a QNP (600 MHz) using CDCl_3_.


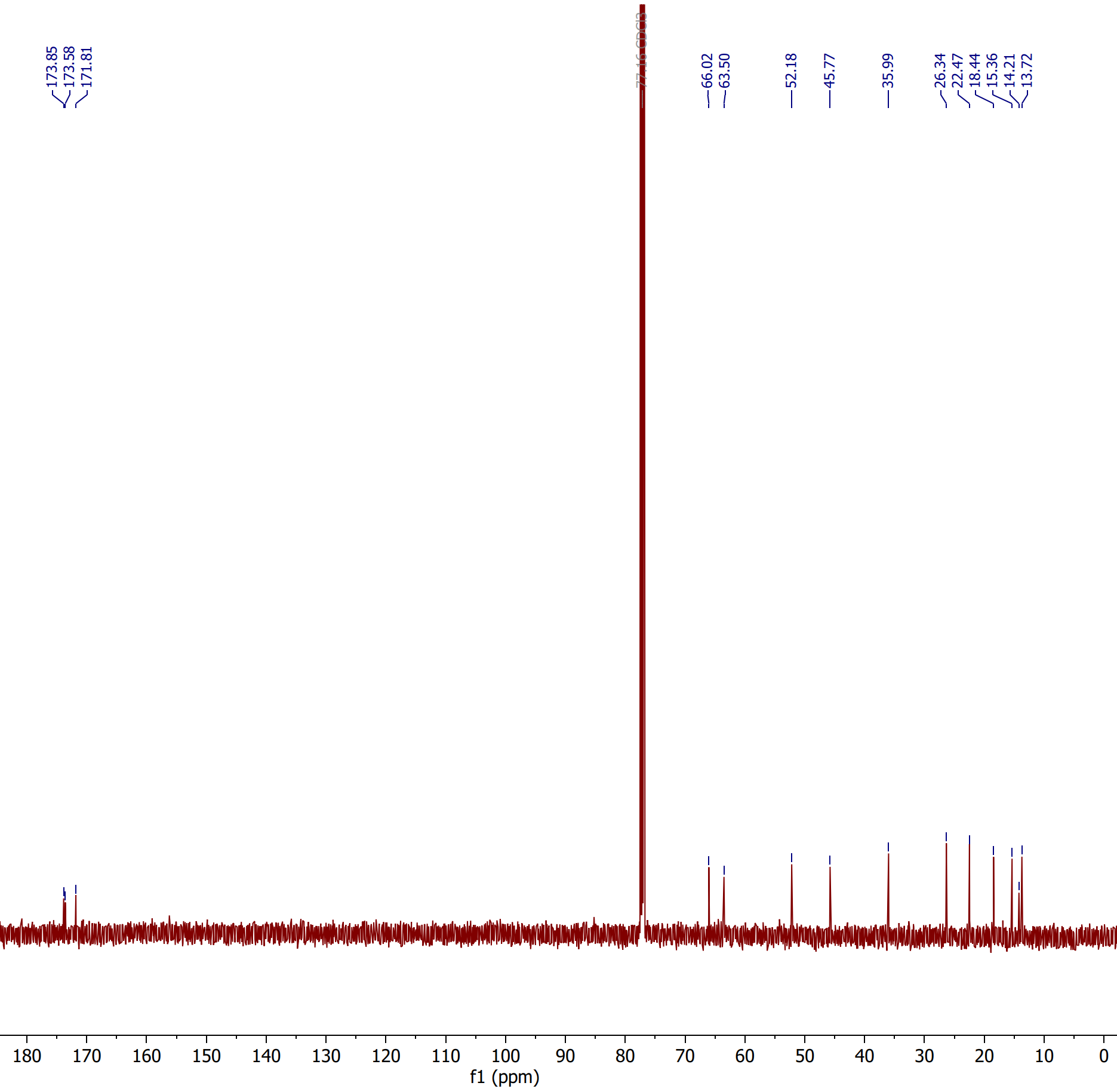


Supplementary Figure 8. ^13^C NMR spectrum of *N*-3-methylbutyryl-*O*-butyryl-L-serine produced by female *Steadoda triangulosa.* The ^13^C NMR spectrum was recorded on a Bruker Avance 600 equipped with a QNP (600 MHz) using CDCl_3_.


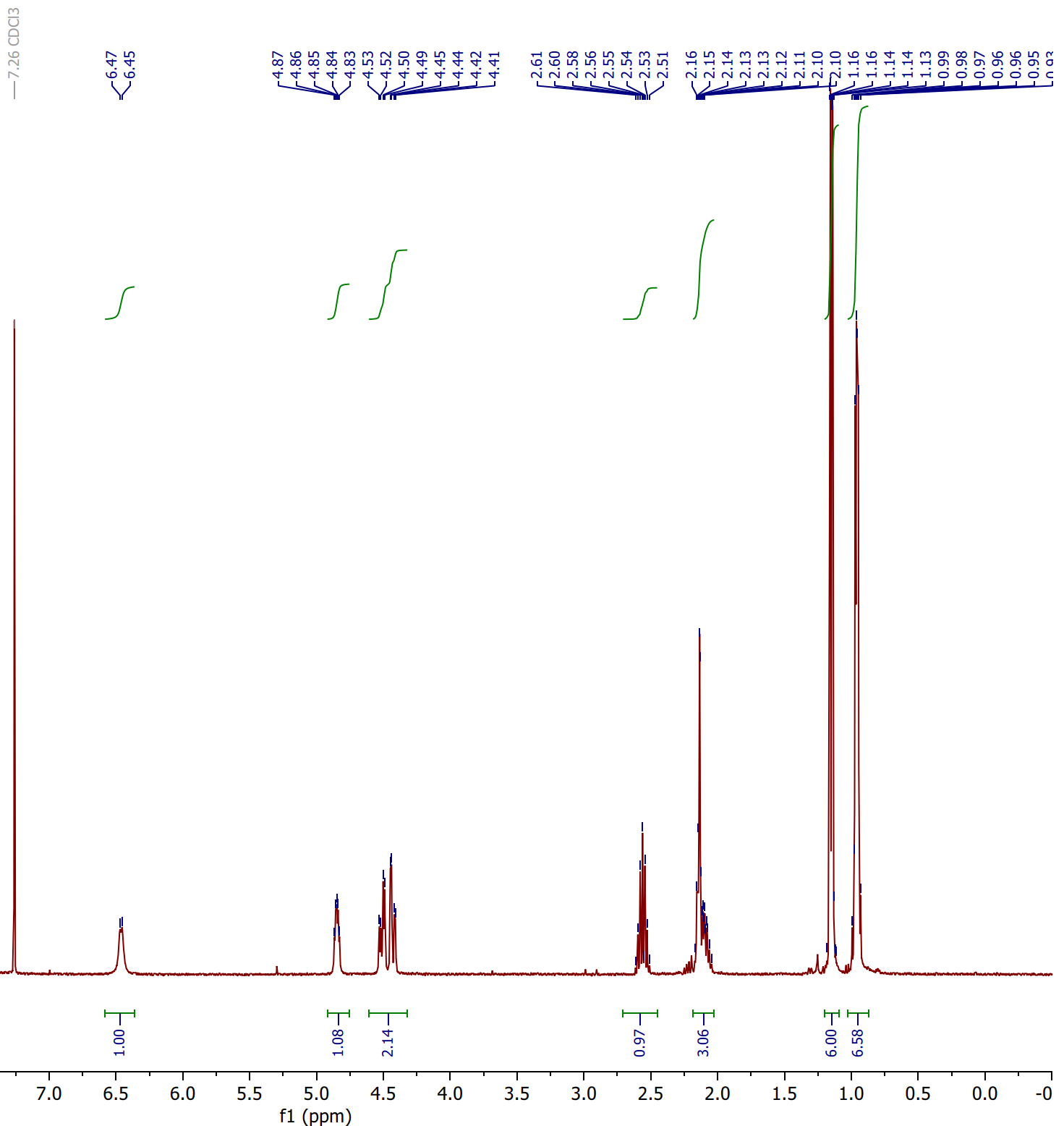


Supplementary Figure 9. ^1^H NMR spectrum of *N*-3-methylbutyryl-*O*-isobutyryl-L-serine produced by female *Steadoda triangulosa.* The ^1^H NMR spectrum was recorded on a Bruker Avance 600 equipped with a QNP (600 MHz) using CDCl_3_.


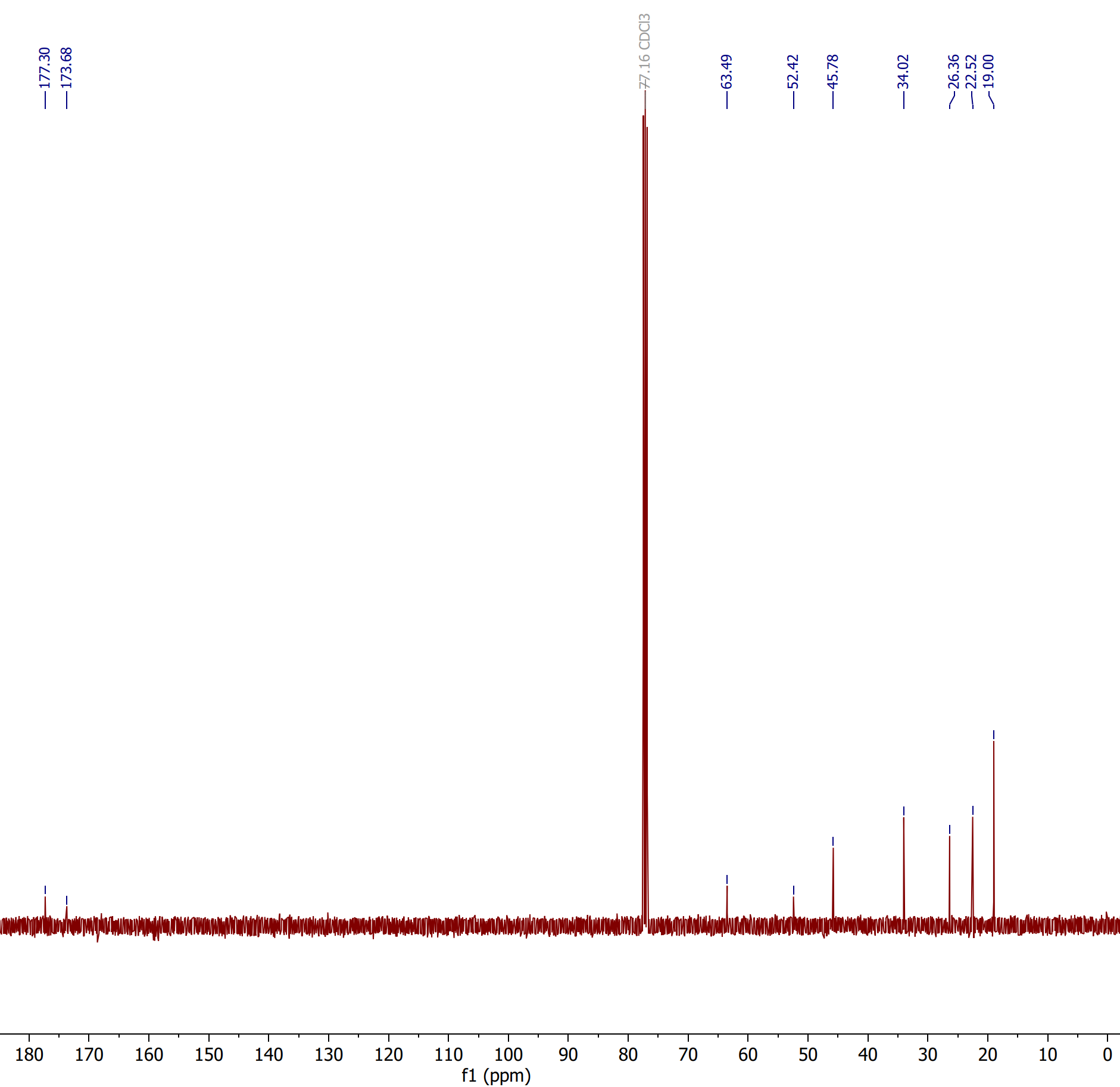


Supplementary Figure 10. ^13^C NMR spectrum of *N*-3-methylbutyryl-*O*-isobutyryl-L-serine produced by female *Steadoda triangulosa.* The ^13^C NMR spectrum was recorded on a Bruker Avance 600 equipped with a QNP (600 MHz) using CDCl_3_.

**Supplementary Methods: Syntheses**

The synthesis of *N*-4-methylvaleroyl-*O*-isobutyroyl-L-serine has already been reported (Fischer et al., 2022), and *N*-3-methylbutyroyl-*O*-isobutyroyl-L-serine and *N*-3-methylbutyroyl-*O*-butyroyl-L-serine were synthesized following the exact protocol detailed in Fischer et al. (2022). The ^1^H and ^13^C NMR spectra of these compounds are reported in Figures S7–10.

**Reference**

Fischer, A., Gries, R., Alamsetti, S.K., Hung, E., Torres, A.C.R., Fernando, S., Meraj, S., Ren, W., Britton, R., Gries, G. (2022) Origin, structure and functional transition of contact sex pheromone components to volatile mate attractant pheromone components in a widow spider. Communication Biology 5, 1156. https://doi.org/10.1038/s42003-022-04072-7
